# Real-world evidence with dapagliflozin in heart failure with reduced ejection fraction in Central Eastern Europe and the Baltic region (EVOLUTION-HF CEE-BA Study)

**DOI:** 10.1093/eschf/xvag085

**Published:** 2026-03-20

**Authors:** Przemysław Leszek, Zoltan Csanádi, Ivan Gruev, Tiina Uuetoa, Diana Žaliaduonytė, Davor Miličić, Kārlis Trušinskis, Jūratė Baukienė, Alexandru Mihai Isvoranu, Ovidiu Chioncel

**Affiliations:** Heart Failure Transplantology and Mechanical Circulatory Support Department, National Institute of Cardiology, Alpejska 42, 04-628 Warsaw, Poland; Department of Cardiology, Faculty of Medicine, University of Debrecen, Debrecen, Hungary; Cardiology Department, National Multiprofile Transport Hospital ‘Tsar Boris III’ Sofia, Sofia, Bulgaria; Cardiology Center, Confido Medical Centre, Tallinn, Estonia; Heart Center, Lithuanian University of Health Sciences, Medical Academy, Kaunas, Lithuania; Heart Center, Kaunas Hospital of Lithuanian University of Health Science, Kaunas, Lithuania; Department of Cardiovascular Diseases, University Hospital Centre Zagreb, University of Zagreb School of Medicine, Zagreb, Croatia; Latvian Center of Cardiology, Pauls Stradiņš Clinical University Hospital, Riga, Latvia; Medical Affairs, AstraZeneca, Vilnius, Lithuania; Medical Affairs, AstraZeneca, Bucharest, Romania; Department Cardiology 1, ‘C.C. Iliescu’ Institute of Cardiovascular Diseases, Bucharest, Romania

**Keywords:** EVOLUTION-HF, Heart failure, Reduced ejection fraction, Dapagliflozin, SGLT2 inhibitor, GDMT

## Abstract

**Introduction:**

Sodium-glucose cotransporter 2 inhibitors (SGLT2i) are currently recommended as one of the four pillars of treatment in heart failure (HF) with reduced ejection fraction (HFrEF). Following the approval of dapagliflozin in 2020, real-world data are relevant for the medical community and payers. This study aimed to characterize the patient population with dapagliflozin initiated for HFrEF in clinical practice in 9 countries from Central Eastern Europe and the Baltic Area (CEE-BA).

**Methods:**

EVOLUTION-HF CEE-BA is a multicentre, multi-country, observational, longitudinal study conducted in 102 centres in Bulgaria, Croatia, Estonia, Hungary, Latvia, Lithuania, Poland, Romania, and Slovenia. All treatment decisions were at the discretion of the patient’s healthcare providers, based on the locally approved product information and routine clinical practice. Patients with type 1 diabetes, prior treatment with dapagliflozin or other SGLT2i, and initiation of dapagliflozin outside the approved HFrEF indication were excluded. The baseline period covered 12 months prior to dapagliflozin initiation, with prospective follow-up continuing up to 12 months or until loss to follow-up, death, or study discontinuation, whichever occurred first. Descriptive statistics and Kaplan–Meier methods were used.

**Results:**

A total of 1131 patients with HFrEF were included in the full analysis set. Ischaemic aetiology was present in 52% of patients. The mean left ventricular ejection fraction was 32%. The most frequent comorbidities were atrial fibrillation (46%), type 2 diabetes (37%), and chronic kidney disease (29%). At the time of dapagliflozin initiation, 93% of patients received any combination of a renin–angiotensin–aldosterone system inhibitor (RAASi), beta-blocker (BB), or mineralocorticoid receptor antagonists (MRA). Of all patients, 60% received concomitantly all three classes plus dapagliflozin for their HFrEF. Except for dapagliflozin, which was administered as 10 mg/day, optimal doses were recorded for 14% for any RAASi, 17% for BB, and 25% for MRA. At 6- and 12-month follow-up, maintenance of dapagliflozin treatment was recorded in 95% and 96% of patients, respectively. The real-world median time to discontinuation of dapagliflozin has not been reached. The percentage of patients receiving all four classes recommended in HFrEF remained stable over the study period. Adverse events were reported spontaneously, as in routine clinical practice in each centre.

**Conclusion:**

This large non-interventional study provides a contemporary perspective of the treatments used in HFrEF over 1-year follow-up. Despite high dapagliflozin persistence rates, a low proportion of patients received complete guideline-directed medical therapy in optimal doses. Improvement of HF management decisions across the CEE-BA region is warranted.

## Graphical Abstract

**Graphical Abstract:**
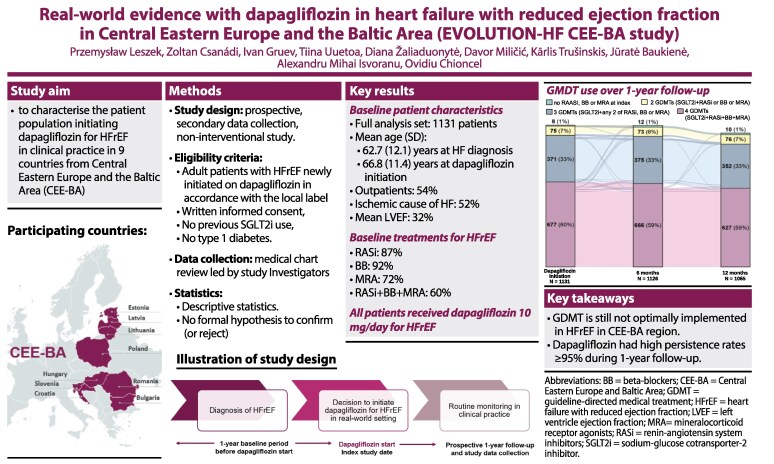
The prospective, non-interventional, international study EVOLUTION-HF CEE-BA included 1131 patients with heart failure and reduced ejection fraction (HFrEF) initiated with dapagliflozin on top of their current treatment. Approximately 60% of patients with HFrEF received guideline-directed medical treatment (GDMT). Persistence with dapagliflozin remained high over 1-year prospective period. The findings are relevant to medical communities across diverse geographies.

## Introduction

Heart failure (HF) is one of the most common cardiovascular (CV) conditions and a growing public health problem.^1^ Both men and women have a lifetime 20%–25% risk of developing the condition.^2,3^ The burden of HF is estimated to be around 64 million worldwide,^4^ with 15 million patients in Europe alone.^5^ Although the HF incidence seems to be stabilizing, the overall prevalence is growing due to ageing and improved survival.^1,6^

Across the spectrum of HF, 50%–60% of patients have HF with reduced ejection fraction (HFrEF) [left ventricular ejection fraction (LVEF) 40% or less].^7–9^ HFrEF remains associated with high rates of morbidity and mortality despite advances in diagnosis and treatment.^10–12^ In general, three areas are critical in the management of HFrEF, specifically, the reduction of avoidable deaths, the reduction of HF-related hospitalizations (hHF), and the improvement of overall quality of life (QoL).^13^ Guideline-directed medical therapy (GDMT) in HFrEF currently includes four main treatment classes: renin–angiotensin system inhibitors (RAASi) [angiotensin-converting enzyme inhibitor (ACE-I), angiotensin receptor blocker (ARB), and angiotensin receptor-neprilysin inhibitor (ARNI)], beta-blockers (BB), mineralocorticoid receptor antagonists (MRA), and sodium-glucose cotransporter-2 inhibitors (SGLT2i).^14^ The recent addition of SGLT2i as the backbone of HFrEF management and the recommendation for ARNI use are based on strong results from clinical trials showing a reduction of mortality and/or hHF.^10,11,15^ Improved clinical outcomes have been observed when all four drug classes are used together (GDMT)^16,17^; however, the rapid simultaneous initiation after diagnosis and titration to the target dose has not become the standard in contemporary clinical practice.^18,19^ Inertia in the adoption of the latest recommendations is observed in the management of HFrEF due to competing factors, including safety concerns, reduced overall compliance with medical recommendations, and/or lack of reimbursement.^13,19–21^

Dapagliflozin is an SGLT2i initially approved to improve glycaemic control in adults with type 2 diabetes (T2D) in addition to diet and exercise.^22,23^ In 2020, based on the results from the Phase III DAPA-HF (Dapagliflozin and Prevention of Adverse-Outcomes in Heart Failure) trial,^10^ the Food and Drug Administration and the European Medicines Agency approved dapagliflozin for patients with HFrEF, with or without T2D.^24–26^ Added to HF standard of care medications, dapagliflozin vs placebo reduced the risk of worsening HF or CV death by 26% (relative risk reduction), and improved symptoms, physical function, and QoL in people with HFrEF.^10,27^

## Study rationale

Following the approval of dapagliflozin in HFrEF, irrespective of T2D, and consolidation as standard of care,^14^ real-world evidence across various geographic areas is becoming more relevant for the medical community and payers. With scarce systematic collection of HF data in Central Eastern Europe and the Baltic Area (CEE-BA) region, there is little evidence on the use of dapagliflozin in HFrEF patients.^28^

EVOLUTION-HF is one of the first multi-national programmes to generate real-world data on the management of patients with HF after hospital discharge^20,21^ and in patients with HFrEF initiating dapagliflozin as part of routine practice.^29,30^ The overall aim of the EVOLUTION-HF CEE-BA study was to describe the patient population with dapagliflozin initiated for HFrEF and to provide early insights into real-world treatment patterns in nine countries (Bulgaria, Croatia, Estonia, Hungary, Latvia, Lithuania, Poland, Romania, and Slovenia).

## Methods

### Study design and setting

This is a multicentre, multi-country, observational, longitudinal study conducted from February 2022 to December 2023 in 102 centres (55 inpatient and 47 outpatient centres) in CEE-BA. Study approvals were obtained from Ethics Committees in all participating countries (Supplementary Table S1). The study was conducted in accordance with the Declaration of Helsinki, the Guidelines for Good Pharmacoepidemiology Practices of the International Society for Pharmacoepidemiology, and local regulations. All patients provided written informed consent to participate in the study before any data collection was performed. All treatment decisions (i.e. treatment initiation, doses, and duration) were at the discretion of the patient’s healthcare providers, based on the locally approved product information and routine practice, and were not mandated by the study protocol.

Inclusion criteria for the EVOLUTION-HF CEE-BA study required a confirmed diagnosis of HFrEF in adult patients (aged ≥18 years) and an interval of 30–60 days between dapagliflozin initiation and enrolment, to ensure a homogeneous population. Patients with type 1 diabetes, prior treatment with dapagliflozin or other SGLT2i, and initiation of dapagliflozin outside the approved label for HF were excluded.

**Figure 1:**
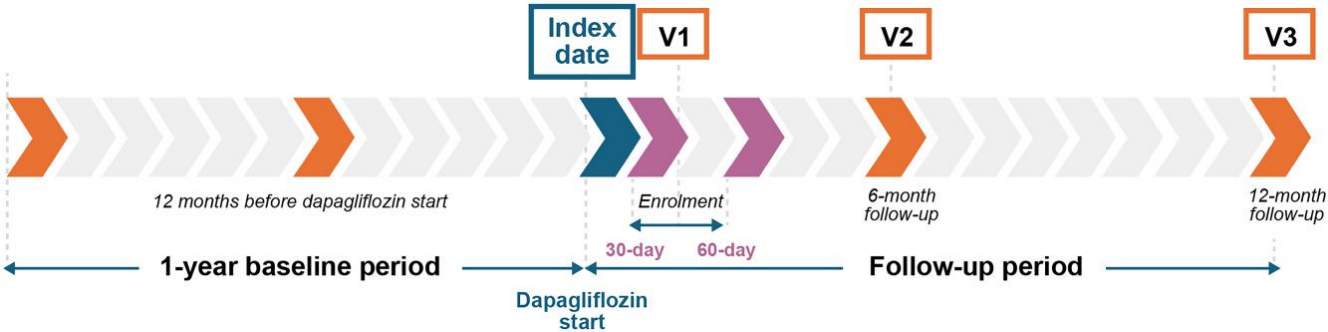
Study design

The baseline period covered 12 months prior to dapagliflozin initiation (index date), and prospective follow-up continued up to 12 months or until loss to follow-up, death, or study discontinuation, whichever occurred first (*Figure 1*). At baseline, study variables included demographics, clinical and laboratory parameters, selected CV and non-CV comorbidities, HFrEF-related history, and treatments used in HFrEF and T2D at the time of the index date. Changes in HF and T2D treatment patterns were recorded at two follow-up assessments: after 6 and 12 months following the dapagliflozin initiation. The protocol approved for the CEE-BA study did not include collecting patient-reported outcomes [the Kansas City Cardiomyopathy Questionnaire (KCCQ)-23 and health-related QoL questionnaires], which were part of the EVOLUTION-HF programme in the United Kingdom, Italy, and Greece.^30^

Study information was collected from patient medical files (paper and/or electronic) and registered by study personnel into a password-protected, pseudo-anonymized, web-based electronic case report form developed specifically for this study. EVOLUTION-HF CEE-BA was an observational, secondary data collection study, where specific reporting and collection of adverse events (AEs) was not required; reporting of any AEs was spontaneous, as per routine clinical practice.

The primary objectives of the study were to describe the baseline demographic and clinical characteristics of patients with newly prescribed dapagliflozin for the treatment of HFrEF, to assess dapagliflozin treatment patterns, including time to discontinuation, as well as to assess patterns of other HF and T2D medication in patients initiated on dapagliflozin for HFrEF.

### Statistical analysis

The study was not designed to confirm (or reject) any predefined hypothesis. No formal sample size was calculated, and the target number of patients to be enrolled was established at country level based on preliminary feasibility assessments and considering the dapagliflozin reimbursement status at study start.

Standard descriptive statistics were applied. The Kaplan–Meier method was used to estimate the median [95% confidence intervals (CI)] time to discontinuation (mTTD) of dapagliflozin, the other HF, and glucose-lowering medications. Patients who did not discontinue treatment during the observation period were censored at their last available observation. In addition, exploratory analyses were conducted using multivariate regression models to evaluate the association between baseline characteristics and discontinuation of dapagliflozin, and subgroup analyses. Subgroups were stratified by type of primary practice (inpatient/outpatient), duration of HF at study entry, and systolic blood pressure at baseline (SBP < or ≥130 mmHg). Statistical analysis was performed using *R* software (https://www-r-project.org/) version RStudio 2023.03.1+446 ‘Cherry Blossom’.

## Results

The total number of subjects enrolled in the EVOLUTION-HF CEE-BA study was 1143, and the full analysis set included 1131 eligible patients. The follow-up data were available for 1126 patients at the 6-month follow-up and for 1065 patients at the 12-month follow-up. A slightly higher proportion of patients in this study were treated in outpatient clinics (53.5%) versus hospital centres (46.5%).

Baseline demographic and clinical characteristics are presented in *Table 1*. The male:female ratio was almost 3:1. The mean age (SD) was 62.7 (12.1) years at HF diagnosis. More than one-third of patients were in the range of 70–80 years at index date.

**Table 1:** Baseline patient characteristics (FAS)

Characteristics	FAS *N* = 1131
Demographic and clinical characteristics^a^	
Age at HF diagnosis, mean (SD), years	62.65 (12.13)
Age at index date, mean (SD), years	66.82 (11.4)
Male, *n* (%)	836 (74%)
BMI, kg/m^2^, *n* (%)	
<18.5	3 (0.27%)
≥18.5 to <25	225 (19.89%)
≥25 to <30	448 (39.61%)
≥30	455 (40.23%)
Blood pressure (BP)	
Systolic BP (SBP), mean (SD), mmHg	126.56 (17.07)
SBP >130 mmHg, *n* (%)	393 (34.75%)
Diastolic BP (DBP), mean (SD), mmHg	77.58 (11.08)
DBP >80 mmHg, *n* (%)	330 (29.18%)
Heart rate, mean (SD), beats/min	76.29 (15.61)
eGFR, mean (SD), ml/min/1.73 m^2^ (*n* = 752)	66.85 (20.99)
eGFR categories, *n* (%)	
15 to < 30 (G4)	19 (2.53%)
30 to < 60 (G3)	278 (36.97%)
60 to < 90 (G2)	319 (42.42%)
≥90 (G1)	136 (18.09%)
uACR categories, mg/g, *n* (%) (*n* = 31)	
<30 (A1)	19 (61.29%)
30–299 (A2)	10 (32.26%)
≥300 (A3)	2 (6.45%)
HbA1c mean (SD), % (*n* = 324)	6.45 (1.19)
≥6%, *n* (%)	192 (59.25%)
NT-proBNP, median (min-max), pg/ml (*n* = 521)	2041 (27.2–35 000)
Principal cause of HF, *n* (%)	
Ischaemic	585 (51.72%)
Non-ischaemic	460 (40.67%)
Unknown	86 (7.60%)
Characteristics of HF^a^	
Time from first diagnosis of HF to index date, median (min-max), months	24.47 (0–533.47)
LVEF, mean (SD), %	32.37 (6.93)
10 to<20%	44 (3.89%)
20 to <30%	257 (22.72%)
30 to <40%	626 (55.35%)
40%	204 (18.04%)
NYHA class, *n* (%)	
II	590 (52.17%)
III	500 (44.21%)
IV	33 (2.92%)
Unknown	8 (0.71%)
Time since discharge of last hHF to index date, median (min-max), days	64.5 (1–412)
Comorbidities^b^	
CV comorbidities, *n* (%)	
Atrial fibrillation	522 (46.15%)
Significant haemodynamic valve insufficiency/stenosis	235 (20.78%)
History of relevant CV conditions and/or ICD/CRT, *n* (%)	
History of coronary revascularization	409 (36.16%)
History of myocardial infarction	372 (32.89%)
History of use of ICD	117 (10.34%)
History of CRT	98 (8.66%)
History of unstable angina	91 (8.05%)
History of stroke	80 (7.07%)
Non-CV conditions	
T2D	414 (36.60%)
CKD	330 (29.18%)
History of COVID-19 infection	131 (11.58%)
History of malignancy	40 (3.54%)
History of psychiatric disorders	22 (1.95%)
Number of comorbidities per patient, median (min-max)	2 (0–8)
CV medication in association with Dapa^c^, *n* (%)	
GDMT^d^	
RAASi	989 (87.44%)
ACE-I	458 (40.50%)
ARB	103 (9.11%)
ARNI	428 (37.84%)
BB	1044 (92.31%)
MRA	815 (72.06%)
RAASi + BB + MRA	677 (59.90%)
Diuretics	858 (75.86%)
Antiplatelets	499 (44.12%)
Anticoagulants	600 (53.05%)
Cardiac glycosides	137 (12.11%)
Vasodilators	59 (5.22%)
If-channel inhibitor	58 (5.13%)
Glucose-lowering drugs, *n* (%)	330 (29.18%)
Biguanides	245 (74.24%)
Insulin	65 (19.69%)
Sulphonylureas	59 (18.87%)
GLP-1 receptor agonists	22 (6.67%)
DPP-4 inhibitors	15 (4.54%)

Percentages are calculated from the FAS number of patients (*n* = 1131), except when otherwise specified. Vericiguat use was reported in 1 patient at index date.

ACE-I, angiotensin-converting enzyme inhibitors; ARB, angiotensin receptor blockers; ARNI, angiotensin receptor-neprilysin inhibitor; BMI, body mass index; CRT, cardiac resynchronization therapy; CKD, chronic kidney disease; CV, cardiovascular; DPP-4, dipeptidyl peptidase 4; eGFR, estimated glomerular filtration rate; FAS, full analysis set; GDMT, guideline-directed medical therapy; GLP-1, glucagon-like peptide-1; HF, heart failure; HbA1c, glycated haemoglobin; ICD, implantable cardioverter defibrillator; LVEF, left ventricular ejection fraction; MRA, mineralocorticoid receptor antagonists; NT-proBNP, *N*-terminal pro hormone BNP; NYHA, New York Heart Association; RAASi, renin–angiotensin–aldosterone system inhibitors; SD, standard deviation; T2D, type 2 diabetes; uACR, urine albumin-to-creatine ratio.

^a^At index date or most recent assessment within 6 months prior to the index date.

^b^In the last 12 months prior to the index date.

^c^At index date.

^d^For GDMT: RAASi group includes ACE-I, ARB, and ARNI. Multiple treatments were recorded in 1048 (92.7%) patients (any combinations) and in 371 (32.8%) patients (any combination of 2 treatments). All treatments are to be considered in association with dapagliflozin.

The median (IQR) time from HF diagnosis to study entry was 24.5 (1.8–78.8) months. Approximately one-third of patients (33.8%) had a recent (<6 months) HF diagnosis, and a similar proportion (30.7%) had an older HF diagnosis (≥5 years), whereas 45.5% of patients were diagnosed with HF ≥6 months to <5 years before enrolment. The main cause of HF was ischaemic heart disease in 51.7% of cases. At index date, most patients were classified as having New York Heart Association (NYHA) II (52.2%) and III (44.2%), with a mean (SD) LVEF of 32.4% (6.9%).

Overall, during the baseline period, 33.4% of patients had at least one hHF. In 32.3% of them, dapagliflozin was initiated either during the most recent hospitalization or after discharge from last hHF, with a median (IQR) time from discharge to dapagliflozin start of 64.5 (8–161) days.

Each patient had a median (IQR) of 2 (1–4) comorbidities (selected). The number of comorbidities increased, whereas renal function decreased with longer HF duration. The most frequent comorbidity of interest reported in the last year before the index date was atrial fibrillation, in around half (46.1%) of the patients included in the EVOLUTION-HF CEE-BA study. Approximately one-third of patients had a concomitant diagnosis of T2D (36.6%) or chronic kidney disease (CKD) (29.2%), with 156 (13.8%) patients presenting both CKD and T2D. The values of estimated glomerular filtration rate (eGFR) were missing in 110 patients (20.9%) enrolled in hospitals and 269 patients (44.5%) enrolled in outpatient clinics. In patients with available eGFR (*N* = 752), a mildly decreased renal function (G2) was reported for most (42.4%).

### Heart failure treatment at index date

All patients included in the study received dapagliflozin 10 mg/day for HFrEF. At index date, most patients (92.7%) had any combination of RAASi (ACE-I, ARB, or ARNI), BB, or MRA, whereas the concomitant use of all the other three GDMTs was recorded in 59.9% of patients (*Table 1*). The use of the four pillars of GDMT (RAASi, BB, MRA, and SGLT2i) was recorded in more patients enrolled in hospital centres, in patients with controlled SBP, and with a longer duration of HF (*Table 2*). Diuretics represented the third most-used therapeutic class (75.8%) received at index date after BB (92.3%) and a RAASi (87.4%). Concomitant use of all four GDMT pillars plus diuretics was reported for 45.1% of patients. Other treatments used for HF are listed in the *Table 1*.

**Table 2:** Key patient and treatment characteristics by subgroups

Characteristic	Type of study centre		SBP at index date		HF duration at index date		
	Hospital	Outpatient	<130 mmHg	≥130 mmHg	<6 months	6 months to <5 years	≥5 years
**Baseline patient characteristics**							
Number of patients	*N* = 526	*N* = 605	*N* = 604	*N* = 527	*N* = 382	*N* = 402	*N* = 347
Age at HF diagnosis, mean (SD), years	61.10 (12.99)	64.01 (11.16)	61.74 (12.84)	63.70 (11.18)	65.90 (11.80)	64.22 (11.15)	57.27 (11.81)
BMI, kg/m^2^, mean (SD)	29.44 (5.31)	29.14 (5.05)	28.78 (5.21)	29.84 (5.06)	29.03 (5.16)	29.29 (5.27)	29.55 (5.06)
Heart rate, mean (SD), beats/min	76.45 (16.42)	76.16 (14.87)	75.87 (15.41)	76.79 (15.83)	78.83 (18.30)	76.59 (14.32)	73.16 (13.11)
eGFR, mean (SD), ml/min/1.73 m^2^	67.52 (20.20) [*N* = 416]	66.03 (21.92) [*N* = 336]	66.52 (21.31) [*N* = 409]	67.25 (20.62) [*N* = 343]	69.67 (20.41) [*N* = 283]	66.55 (22.16) [*N* = 250]	63.55 (19.89) [*N* = 219]
HbA1c mean (SD), % (*n* = 179)	6.23 (1.04) [*N* = 208]	6.80 (1.43) [*N* = 117]	6.40 (1.16) [*N* = 179]	6.47 (1.31) [*N* = 146]	6.55 (1.40) [*N* = 111]	6.46 (1.07) [*N* = 103]	6.29 (1.18) [*N* = 111]
No. of comorbidities per patient, median (min-max)	2 (0–7)	3 (0–8)	2 (0–8)	2 (0–8)	2 (0–7)	2 (0–8)	3 (0–8)
**Baseline GDMT use** ^a^							
ACE-I, *n* (%)	197 (37.45%)	261 (43.14%)	215 (35.6%)	243 (46.11%)	169 (44.24%)	138 (34.33%)	151 (43.52%)
ARB, *n* (%)	45 (8.56%)	58 (9.59%)	44 (7.28%)	59 (11.20%)	35 (9.16%)	34 (8.46%)	34 (9.80%)
ARNI, *n* (%)	228 (43.35%)	203 (33.55%)	253 (41.89%)	178 (33.78%)	125 (32.72%)	171 (42.54%)	135 (38.90%)
BB, *n* (%)	493 (93.73%)	551 (91.07%)	566 (93.71%)	478 (90.70%)	341 (89.27%)	373 (92.79%)	335 (95.10%)
MRA, *n* (%)	402 (76.43%)	413 (68.26%)	461 (76.32%)	354 (67.17%)	283 (74.08%)	293 (72.89%)	239 (68.88%)
RAASi + BB + MRA*, *n* (%)	337 (64.06%)	340 (56.19%)	381 (63.07%)	296 (56.16%)	227 (59.42%)	238 (59.20%)	212 (61.09%)
**6-month GDMT use** ^a^							
Number of patients	*N* = 523	*N* = 603	*N* = 600	*N* = 526	*N* = 381	*N* = 400	*N* = 345
ACE-I, *n* (%)	185 (35.37)	258 (42.79%)	209 (34.83%)	234 (44.49%)	164 (43.04%)	132 (33.00%)	147 (42.61%)
ARB, *n* (%)	44 (8.41%)	55 (9.12%)	42 (7.00%)	57 (10.84%)	32 (8.40%)	35 (8.75%)	32 (9.28%)
ARNI, *n* (%)	241 (46.08%)	206 (34.16%)	258 (43.00%)	189 (35.93%)	132 (34.65%)	173 (43.25%)	142 (41.16%)
BB, *n* (%)	490 (93.69%)	548 (90.88%)	560 (93.33%)	478 (90.87%)	342 (89.76%)	372 (93.00%)	324 (93.91%)
MRA, *n* (%)	392 (74.95%)	410 (67.99%)	452 (75.33%)	350 (66.54%)	278 (72.97%)	290 (72.50%)	234 (67.83%)
RAASi + BB + MRA*, *n* (%)	328 (62.71%)	336 (55.72%)	375 (62.50%)	291 (55.32%)	210 (55.11%)	239 (59.75%)	207 (60.00%)
**12-month GDMT use** ^a^							
Number of patients	*N* = 490	*N* = 575	*N* = 564	*N* = 501	*N* = 361	*N* = 379	*N* = 325
ACE-I, *n* (%)	169 (34.49%)	242 (42.09%)	191 (33.87%)	220 (43.91%)	152 (42.11%)	127 (33.51%)	132 (40.62%)
ARB, *n* (%)	41 (8.37%)	53 (9.22%)	40 (7.09%)	54 (10.78%)	30 (8.31%)	33 (8.71%)	31 (9.54%)
ARNI, *n* (%)	232 (47.35%)	200 (34.78%)	252 (44.68%)	180 (35.93%)	131 (36.29%)	163 (43.01%)	138 (42.46%)
BB, *n* (%)	455 (92.86%)	524 (91.13%)	526 (93.26%)	453 (90.42%)	322 (89.20%)	351 (92.61%)	306 (94.15%)
MRA, *n* (%)	364 (74.29%)	389 (67.65%)	422 (74.82%)	331 (66.07%)	260 (72.02%)	276 (72.82%)	217 (66.77%)
RAASi + BB + MRA*, *n* (%)	306 (62.44%)	321 (55.82%)	355 (62.94%)	272 (54.29%)	212 (58.72%)	222 (58.57%)	193 (59.38%)
**12-month dapagliflozin discontinuation rate**							
Rate per 100 patient-years (95% CI)	10.79 (8.14–13.44)	6.30 (4.36–8.24)	9.55 (7.2–11.89)	7.05 (4.86–9.23)	8.16 (5.42–10.91)	8.54 (5.81–11.28)	8.42 (5.5–11.34)

ACE-I, angiotensin-converting enzyme inhibitors; ARB, angiotensin receptor blockers; ARNI, angiotensin receptor-neprilysin inhibitor; BMI, body mass index; eGFR, estimated glomerular filtration rate; GDMT, guideline-directed medical therapy; HF, heart failure; HbA1c, glycated haemoglobin; MRA, mineralocorticoid receptor antagonists; *N*, number; RAASi, renin–angiotensin–aldosterone system inhibitors; SD, standard deviation.

^a^All patients included in the study had treatment with Dapa 10 mg/day for heart failure.

Doses of GDMTs (except dapagliflozin, which was administered as 10 mg/day in all cases) are listed in Supplementary Table S2. At index date, use of GDMT optimal doses recommended by the 2021 ESC Guidelines [14] was observed in 13.5% of patients for any RAASi, 17.1% for BB, and 25.1% for MRA (Supplementary Figure S1). Overall, a higher proportion of patients used MRA in optimal and high dose (50%–99% of the optimal dose), followed by BB and RAASi. Of all RAASi users at index date (*n* = 989), 15.5% and 26.9% received the treatment in optimal dose and high dose, respectively.

### Glucose control and glucose-lowering treatment

Baseline glycated haemoglobin (HbA1c) (measured at index date or in the last 6 months before index date) was available for almost one-third of patients (29%) (*Table 1*). In total, 330 patients (confirmed with T2D) were receiving glucose-lowering treatments at the time of dapagliflozin start for HFrEF, mostly biguanides (74.2%) (*Table 1*).

### Heart failure treatment changes over 1-year follow-up

**Figure 2:**
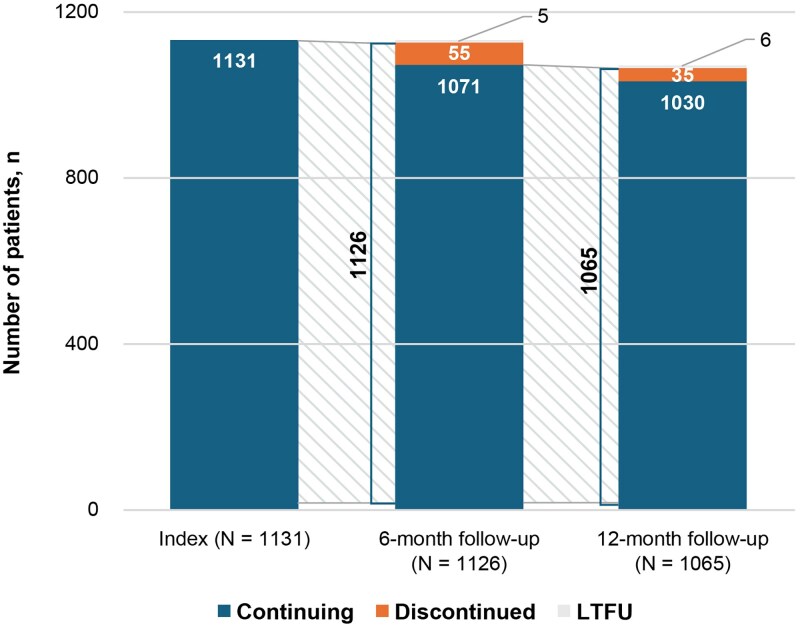
Dapagliflozin attrition rates during the study (1-year follow-up). LTFU, lost to follow-up

**Figure 3:**
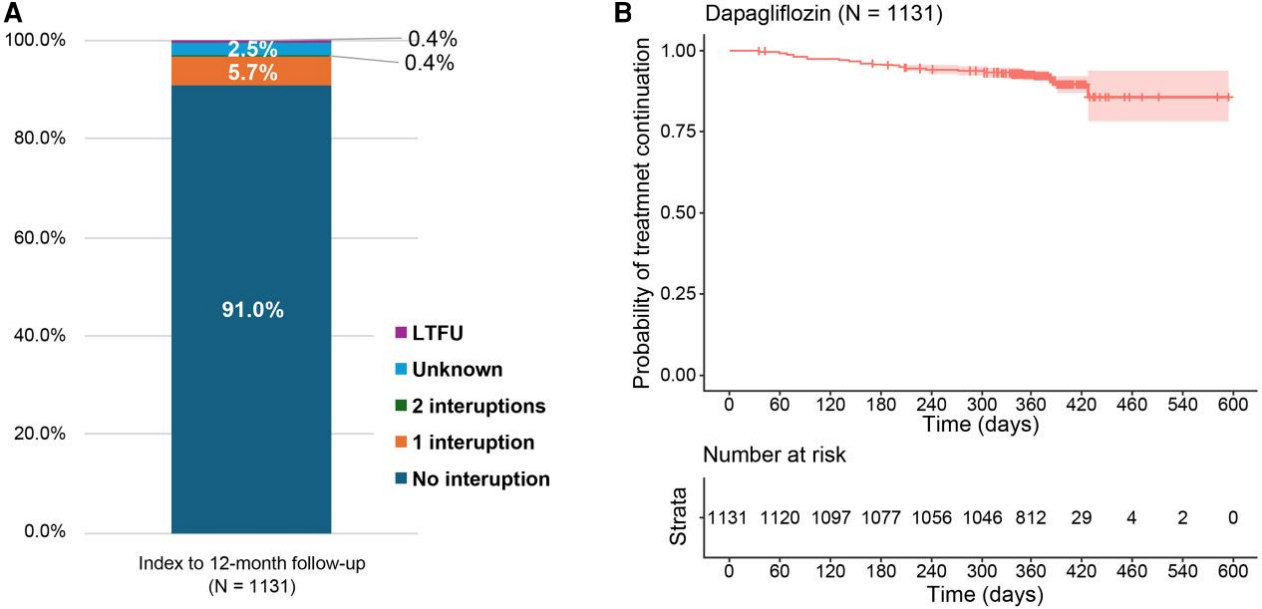
Dapagliflozin treatment patterns during the study (1-year follow-up). Cases of dose interruptions and restart (*A*) and time to dapagliflozin discontinuation (*B*). LTFU, lost to follow-up

The attrition rates of dapagliflozin between index and 6-month follow-up, and from 6-month to 12-month follow-up were 5.3% and 3.8%, respectively. Overall, 8.9% of patients discontinued the study (*Figure 2*). At 6- and 12-month follow-up, maintenance of dapagliflozin was recorded in 95.0% and 96.0% of patients, respectively. During the study, dapagliflozin interruptions were recorded in 73 instances, with a total of 44 cases where dapagliflozin was not restarted (*Figure 3A*). Overall, 94 discontinuations (8.3%) of treatment with dapagliflozin were recorded during the study, with a discontinuation rate of 8.4 per 100 patient-years (95% CI: 6.7–10.0). The real-world mTTD of dapagliflozin has not been reached (*Figure 3B*). Across subgroups, the dapagliflozin discontinuation rate per 100 patient-years ranged from 6.3 (95% CI: 4.30–8.24) in outpatients and 10.8 (95% CI: 8.14–13.44) in hospital study sites (*Table 2*).

**Figure 4:**
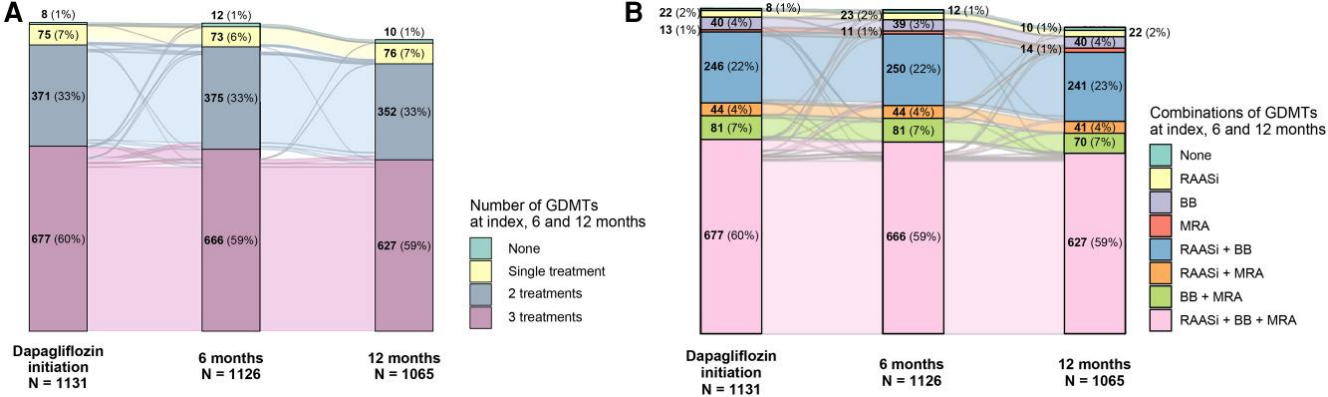
High-level changes (alluvial plot) of the number of GDMT drugs for HF that were associated with dapagliflozin (FAS) (*A*) and high-level changes (alluvial plot) of GDMT combinations associated with dapagliflozin (FAS) (*B*). GDMT, guideline-directed medical treatment; HF, heart failure

During the 1-year follow-up, the percentage of patients receiving any of the GDMTs (irrespective of class or combination of classes) plus dapagliflozin remained stable (92.7% at index date, 92.5% at the 6-month follow-up and 91.9% at the 12-month follow-up). The percentage of patients receiving all four GDMT classes recommended in HFrEF also remained stable over 1-year (59.9% at index, 59.1% at the 6-month follow-up and 58.9% at the 12-month follow-up) (*Figure 4A* and *B*). Except for dapagliflozin, which was used as 10 mg/day, treatment changes consisting of any dose changes, additions, or discontinuations were recorded in a low number of cases (Supplementary Table S3). Adjustments reported for at least 5% of patients reaching the 6- and 12-month follow-up were mentioned only for diuretics, ARNI and BB. The percentage of patients with other treatments for HF, as well as other CV medication also remained stable across the 1-year follow-up [data not shown].

In the overall study cohort, 414 patients (36.6%) had T2D and at index date. T2D treatment changes over time were infrequent and mainly involved the initiation or discontinuations of insulin. Non-SGLT2i glucose-lowering agents were received by 330 patients (29.2%) at index date, 333 (29.6%) at 6-month, and 314 (29.5%) at 12-month follow-up, reflecting a trend of stability during the prospective phase of the study.

### Association between baseline characteristics and dapagliflozin discontinuation

**Figure 5:**
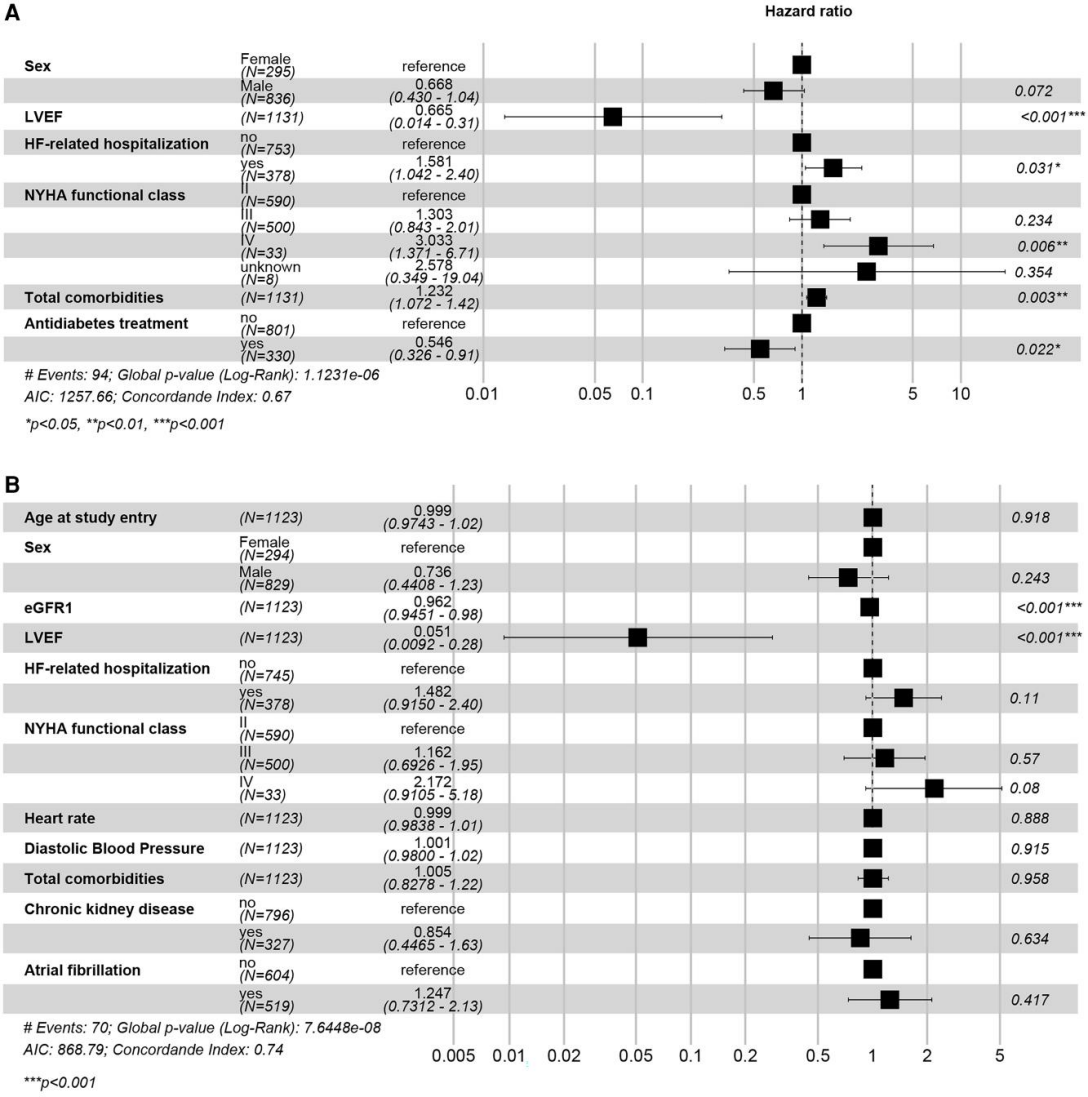
Best-performing Cox model (AIC criteria) with (*A*) and without (*B*) statistically significant predictors for dapagliflozin discontinuation based on selected clinical characteristics. AIC, Akaike information criterion; eGFR, estimated glomerular filtration rate; HF, heart failure; LVEF, left ventricular ejection fraction; NYHA, New York Heart Association

An unadjusted Cox proportional hazards model with selected baseline clinical characteristics indicated that eGFR (HR = 0.96, 95% CI 0.95–0.97, *P* < .001), LVEF (HR = 0.05, 95% CI 0.01–0.21, *P* < .001), NYHA IV (HR = 4.09, 95% CI 1.91–8.8, *P* < .0001), hHF (HR = 1.84, 95% CI 1.23–2.76, *P* = .003), comorbidities (HR = 1.18, 95% CI 1.04–1.35, *P* = .012), CKD (HR = 2.02, 95% CI 1.35–3.04, *P* = .001), and heart rate (HR = 1.01, 95% CI 1–1.02, *P* = .024) were associated with dapagliflozin discontinuation. The best-performing model, including only statistically significant predictors, showed the same baseline clinical factors (LVEF, NYHA IV, hHF, antidiabetic treatment, and comorbidities) were associated with dapagliflozin discontinuation (*Figure 5A*). However, in the adjusted models, run including all selected clinical characteristics, only eGFR and LVEF remained significant baseline characteristics associated with dapagliflozin discontinuation (*P* < .001 for both) (*Figure 5B*).

## Discussion

To our knowledge, EVOLUTION-HF is the first non-interventional, longitudinal study conducted in the HFrEF population initiating dapagliflozin in real-life settings in the CEE-BA region. The study provided a detailed overview of the characteristics of a large cohort (*N* = 1131) with HFrEF with recent initiation and 1-year use of dapagliflozin. Moreover, the study results include information on the contemporary use of GDMTs and other HF treatments, as well as glucose-lowering drugs in the HFrEF population.

The baseline characteristics of this cohort are consistent with those observed in HFrEF populations in other studies.^31–33^ We noticed a high prevalence of comorbid conditions, particularly atrial fibrillation (46.2%), T2D (36.6%), and CKD (29.2%). It is worth noting that more than one-third of all patients with HFrEF missed the eGFR values recorded in the medical charts in cardiology clinics in the last year before the initiation of dapagliflozin. The non-cardiac comorbidities may play a role in preventing complete GDMT and up-titration to optimal doses in clinical practice, although the clinical trials did not show significant interactions.^34^ EVOLUTION-HF provides an image of the multimorbidity burden across CEE-BA countries and the importance of a comprehensive approach to managing HF and associated conditions. We therefore highlight the value brought by interventions that can effectively address multiple conditions, such as SGLT2i with proven benefits in cardio-reno-metabolic conditions.

### Use of heart failure treatments

At study index (initiation of the SGLT2i, Dapa 10 mg/day for HFrEF), most patients had treatment with RAASi, BB, and/or MRA (87.4%, 92.3%, and 72.1%, respectively). Of RAASi users, almost 40% of patients had treatment with an ACE-I and/or ARNI (sacubitril/valsartan), and 10% with an ARB. The situation is not different from other European countries participating in the EVOLUTION-HF programme, except for the use of sacubitril/valsartan at index date, with higher percentages in the UK (54%), Portugal (55%), and Italy (72%) in a similar patient population.^30^ One important barrier to ARNI prescription in the CEE-BA region is the administrative burden with requirements imposed by national protocols for drug reimbursement. A steady increase in ARNI prescription has been observed in our study and other real-world analyses.^35,36^

Complete GDMT strategy for HFrEF (with ACE-I/ARNI or ARB in case of intolerance, BB and MRA, plus SGLT2i [dapagliflozin])^14^ was recorded in 60% of patients, potentially reflecting the ongoing phase of the transition to full adoption of guidelines into clinical practice in the CEE-BA region at the time of the study, as well as the fragmentation of care between inpatient and outpatient care. It is still debated whether the optimal way to achieve the four-pillar GDMT is during hospitalization or in an outpatient setting.^37,38^ In our study, a higher proportion of patients enrolled in hospital centres than outpatient clinics received complete GDMT from index date and during the study (*Table 2*). We consider that the multidisciplinary care available in hospitals is a valuable resource for the initiation and up-titration of doses, with clear recommendations at discharge on the frequency of outpatient follow-ups.

In general, over the 1-year follow-up, the proportion of patients receiving each of the mainstay classes used for the HF treatment remained stable. These findings are similar to those from the Italian EVOLUTION-HF cohort (66%)^29^ and other observational data (58%)^39^; however, the proportion remains unsatisfactory.

At index date, a low percentage of patients with HFrEF in the CEE-BA region received RAASi, BB, and MRA in optimal doses, as listed in the ESC 2021 Guidelines.^14^ Taken together, ∼15% and 20% of all patients had an optimal or high dose (50%–90% of the optimal dose) of ACE-I and ARNI, respectively, at the time of initiating treatment with dapagliflozin. Moreover, the subgroup analyses showed that almost 50% of patients did not have SBP controlled at index date, with a reduced percentage of GDMT use in this subgroup. Given that HF treatments, including GDMTs, were implemented at the discretion of treating physicians, reflecting the routine clinical practice, the results clearly indicate the unachieved potential of pharmacological management, particularly related to guideline-recommended target doses. Furthermore, during the subsequent 12-month follow-up, GDMT use and target doses remained relatively stable, which we consider to be an indicator of therapeutic inertia. While most patients received lower than target doses and blood pressure was not adequately controlled, additional strategies and more efforts are needed to optimize the HF pharmacological treatment, including simultaneous initiation of all GDMTs and rapid up-titration.^14^ In this context, an advantage for dapagliflozin is that the usual dose of 10 mg/day is the optimal dose.

The reduced discontinuation rate of dapagliflozin over 1-year (<10%), with mTTD not achieved during the study follow-up, irrespective of the HF duration, is encouraging, holding practical implications for the medical community in the CEE-BA region. A similar result was found in the cohort from the United Kingdom.^30^ A much higher rate (>20%) has been published in patients initiating dapagliflozin within 12 months of a hHF.^21^ In exploratory analyses performed in the EVOLUTION-HF CEE-BA study, several clinical characteristics (e.g. eGFR, LVEF, NYHA IV, hHF, comorbidities) were found to be associated with discontinuation of dapagliflozin treatment. We did not assess the causality of each variable with a treatment pattern, therefore, no correlation between increasing or decreasing LVEF or eGFR with dapagliflozin discontinuation can be concluded. Nevertheless, adherence is a crucial aspect for patients’ outcomes. Maintenance of treatments with proven benefits, such as SGLT2i, may contribute to the reduction of hHF and mortality rates in HFrEF.^10–14^

Considering the limited epidemiological information available and the historically lower access to novel therapies in the CEE-BA region than in the Western world,^28^ the understanding of actual use of HFrEF pharmacotherapy is crucial to improve the HF strategy and patients’ outcomes.

### Limitations

Due to the observational/non-interventional nature of the study and the inherent characteristics of such a design, findings in the EVOLUTION-HF CEE-BA study may be subject to bias, such as selection bias (particularly due to differences in reimbursement across countries), limitations in data availability, and variability in local treatment practices and guidelines. All these factors have impacted the generalizability of the findings. The variables collected in the study did not include the start date of the concomitant HF medications, any laboratory parameters, vital signs, or hospitalizations over 1-year follow-up, nor dapagliflozin discontinuation rates due to lack of reimbursement (Supplementary Table S4). Hence, the study cannot inform on dapagliflozin treatment effectiveness, only on treatment patterns. Another limitation is related to the variability of GDMT reimbursement criteria across participating countries, impacting the overall use of these medications in clinical practice, and thus the results related to HF treatment in the study. Difficulties in enrolling patients in some countries due to lack of dapagliflozin reimbursement at study start did not impact the sample size, given the descriptive character of the study. Dapagliflozin discontinuation rates related to lack of reimbursement were not collected.

The routine laboratory indicators (NT-proBNP, eGFR, HbA1c, and urinary albumin-creatinine ratio) were missing to a higher extent, a finding that requires further investigation in each country to better understand the reasons behind it. Lastly, due to its secondary data collection design without study-specific AEs acquisition, no safety assessment could be carried out.

The real-world nature of this study is a limitation, but it could also be seen as an advantage. Collecting data in a comprehensive, standardized manner across multiple centres and countries allowed for the consolidation of real-world evidence on clinical characteristics and treatments used in a large HFrEF patient population. Depicting the real-life settings in various healthcare settings in the region, the EVOLUTION-HF CEE-BA study provides valuable insights into the treatment patterns in routine clinical practice.

## Conclusions

EVOLUTION-HF CEE-BA is the first large non-interventional programme collecting data on the use of dapagliflozin in patients with HFrEF across different healthcare systems and socio-economic conditions. The study revealed the slow adoption of all four pillars of GDMT in both hospital and ambulatory settings, with overall stability of the treatment pattern over a 1-year follow-up. The clinical inertia may have different and complex reasons that deserve to be understood and addressed.

## Supplementary Material

xvag085_Supplementary_Data

## Declarations

### Data Availability

No data were generated or analysed for this manuscript.

### Pre-registered Clinical Trial Number

None supplied.
