# Supplementary material for: Real-world evidence with dapagliflozin in heart failure with reduced ejection fraction in Central Eastern Europe and the Baltic region (EVOLUTION-HF CEE-BA Study)
Source: ESC Heart Fail. 2026 Mar 20;13(3):xvag085. doi: 10.1093/eschf/xvag085 (PMC13175253; doi:10.1093/eschf/xvag085)
Supplement: xvag085_Supplementary_Data [file xvag085_supplementary_data.zip › Supplementary Figure 1.docx]

- **Supplementary Figure 1. Overview of GDMT dose categories at index date by treatment class (FAS)**
  - *Legend***:** ACE-I, angiotensin conversion enzyme inhibitors; ARB, angiotensin-receptor blockers; ARNI, angiotensin receptor-neprilysin inhibitor; BB, beta-blockers; MRA, mineralocorticoid receptor antagonist.
  - **Note**: Small dose=up to 49% of optimal dose; High dose=50-99% of optimal dose; Optimal dose=100 or >100%; None=No drug within that class; Other or missing=other drugs within that class not listed in the ESC 2021 Guidelines.
- **Supplementary Table 1. List of Ethics Committees study approval/favourable opinion numbers in participating countries**

| Country | Ethics Committee Name | Approval (number and date) |
| --- | --- | --- |
| Bulgaria | Ethics Committee for Clinical Trials  (Етична комисия по клинични  изпитвания) | EKKи 0030/19-Jan-2022 |
| Croatia | Central Ethics Commission (SEP) | 381-14-09/21-21-04 from 04-  Jan-2022 |
| Estonia | Research Ethics Committee of the  University of Tartu (UT REC) | 356/T-10 from 20-Dec-2021 |
| Hungary | Medical Reaserch Council (Egeszsegugyi  Tudomanyos Tanacs ETT TUKEB) | BMEU/187-3/2022/EKU (13-Jul-  2022) and BMEU/187-5/2022/EKU  (08-Sep-2022) |
| Latvia | Ethics Committee for Clinical Research  at Pauls Stradins Clinical University  Hospital Development Society | 240522 - 1E from 24-May-  2022 |
| Lithuania | Lietuvos bioetikos komitetas  (Lithuanian BioEthics Committee) | L-22-04/1 from 28-Apr-2022 |
| Poland | Terenowa Komisja Bioetyczna przy  Narodowym Instytucie Kardiologii | IK.NPIA.0021.29.1964/22 of  15-Mar-2022 |
| Romania | Comisia Națională de Bioetică a  Medicamentului și Dispozitivelor  Medicale (National BioEthics Committee  for Medicines and Medical Devices) | 13SNI / 16-Dec-2021 |
| Slovenia | Komisija Republike Slovenje za  medicinsko etiko | 0120-499/2021/3 from 7-Dec-  2021 and 0120-499/2021/6  from 21-Jan-2022 |

- **Supplementary Table 2**. **Distribution of doses of GDMTs excluding dapagliflozin at index date**

| **Class or Agent** | **Dose/day** | | **Number of patients (%)** |
| --- | --- | --- | --- |
| **ACE-I** | | | |
| Enalapril | | n (%) | |
| 1.25 mg/day  5 mg/day  10 mg/day  20 mg/day | | 1 (10.00%)  1 (10.00%)  5 (50.00%)  3 (30.00%) | |
| *2021 ESC Guidelines recommendations: starting dose 2.5 mg b.i.d., target dose 10-20 mg b.i.d* | | | |
| Lisinopril | | n (%) | |
| 2.5 mg/day  5 mg/day  10 mg/day  15 mg/day  20 mg/day  40 mg/day | | 1 (5.00%)  2 (10.00%)  11 (55.00%)  1 (5.00%)  4 (20.00%)  1 (5.00%) | |
| *2021 ESC Guidelines recommendations: starting dose 2.5 mg o.d., target dose 20-35 mg o.d.* | | | |
| Ramipril | | n (%) | |
| 1.25 mg/day  2.5 mg/day  3 mg/day  3.75 mg/day  5 mg/day  7 mg/day  7.5 mg/day  10 mg/day  12.5 mg/day  20 mg/day 1 | | 13 (4.94%)  85 (32.32%)  1 (0.38%)  1 (0.38%)  105 (39.92%)  1 (0.38%)  2 (0.76%)  53 (20.15%)  1 (0.38%)  (0.38%) | |
| *2021 ESC Guidelines recommendations: starting dose 2.5 mg o.d., target dose 10 mg o.d.* | | | |
| Perindopril* | | n (%) | |
| 2 mg/day  2.5 mg/day  4 mg/day  5 mg/day  6 mg/day  7.5 mg/day  8 mg/day  9 mg/day  10 mg/day  12 mg/day | | 5 (3.60%)  24 (16.66%)  11 (7.91%)  53 (36.80%)  1 (0.72%)  2 (1.44%)  12 (8.63%)  1 (0.72%)  34 (23.61%)  1 (0.72%) | |
| Zofenopril* | | n (%) | |
| 3.75 mg/day  7.5 mg/day  15 mg/day  30 mg/day  60 mg/day | | 1 (5.00%)  12 (60.00%)  1 (5.00%)  1 (5.00%)  5 (25.00%) | |
| Quinapril* | | n (%) | |
| 10 mg/day | | 1 (100%) | |
| **ARB** | | | |
| Candesartan | | n (%) | |
| 4 mg/day  6 mg/day  8 mg/day  16 mg/day  24 mg/day  32 mg/day | | 4 (10.00%)  1 (2.50%)  16 (40.00%)  12 (30.00%)  1 (2.50%)  6 (15.00%) | |
| *2021 ESC Guidelines recommendations: starting dose 4 mg o.d., target dose 32 mg o.d.* | | | |
| Valsartan | | n (%) | |
| 40 mg/day  60 mg/day  80 mg/day  100 mg/day  160 mg/day  240 mg/day  320 mg/day | | 5 (17.86%)  1 (3.57%)  10 (35.71%)  1 (3.57%)  9 (32.14%)  1 (3.57%)  1 (3.57%) | |
| *2021 ESC Guidelines recommendations: starting dose 40 mg b.i.d., target dose 160 mg b.i.d.* | | | |
| Losartan | | n (%) | |
| 25 mg/day  100 mg/day | | 1 (50.00%)  1 (50.00%) | |
| *2021 ESC Guidelines recommendations: starting dose 50 mg o.d., target dose 150 mg o.d.* | | | |
| Telmisartan* | | n (%) | |
| 40 mg/day  80 mg/day  160 mg/day | | 7 (31.82%)  14 (63.64%)  1 (4.55%) | |
| Olmesartan* | | n (%) | |
| 20 mg/day  40 mg/day | | 3 (37.50%)  5 (62.50%) | |
| Irbesartan* | | n (%) | |
| 150 mg/day  300 mg/day | | 1 (33.33%)  2 (66.67%) | |
| **ARNI** | |  | |
| Sacubitril/valsartan | | n (%) | |
| 24/26 mg o.d.  24/26 mg b.i.d.  49/51 mg o.d.  49/51 mg b.i.d.  49/51 mg t.i.d.  73/77 mg o.d.  73/77 mg b.i.d.  97/103 mg o.d.  97/103 mg b.i.d. | | 19 (4.41%)  170 (39.44%)  23 (5.34%)  117 (27.15%)  1 (0.23%)  1 (0.23%)  1 (0.23%)  10 (2.32%)  89 (20.65%) | |
| *2021 ESC Guidelines recommendations: starting dose 49/51 mg b.i.d. (24/26 mg b.i.d. in selected patients), target dose 97/103 mg b.i.d* | | | |
| **Beta-blockers** | |  | |
| Bisoprolol | | n (%) | |
| 1.25 mg/day  2 mg/day  2.5 mg/day  3.75 mg/day  5 mg/day  7.5 mg/day  10 mg/day  12.5 mg/day  15 mg/day  20 mg/day | | 20 (3.95%)  1 (0.20%)  107 (21.15%)  8 (1.58%)  215 (42.49%)  31 (6.13%)  108 (21.34%)  1 (0.20%)  9 (1.78%)  6 (1.19%) | |
| *2021 ESC Guidelines recommendations: starting dose 1.25 mg o.d., target dose 10 mg o.d.* | | | |
| Carvedilol | | n (%) | |
| 3.125 mg/day  6.25 mg/day  12.5 mg/day  13 mg/day  18.625 mg/day  18.75 mg/day  25 mg/day  37.5 mg/day  50 mg/day  62.5 mg/day  100 mg/day | | 1 (0.65%)  17 (11.11%)  42 (27.45%)  1 (0.65%)  1 (0.65%)  5 (3.27%)  41 (26.80%)  11 (7.19%)  32 (20.92%)  1 (0.65%)  1 (0.65%) | |
| *2021 ESC Guidelines recommendations: starting dose 3.125 mg b.i.d., target dose 25 mg b.i.d. (target dose 50 mg b.i.d. if >85 kg).* | | | |
| Nebivolol | | n (%) | |
| 1.25 mg/day  2.5 mg/day  5 mg/day  7.5 mg/day  10 mg/day | | 2 (2.20%)  20 (21.98%)  62 (68.13%)  1 (1.10%)  6 (6.59%) | |
| *2021 ESC Guidelines recommendations: starting dose 1.25 mg o.d., target dose 10 mg o.d.* | | | |
| Metoprolol succinate (CR/XL) | | n (%) | |
| 12.5 mg/day  23.75 mg/day  25 mg/day  47.5 mg/day  50 mg/day  62.5 mg/day  71.25 mg/day  75 mg/day  95 mg/day  100 mg/day  125 mg/day  142.5 mg/day  150 mg/day  200 mg/day  225 mg/day  250 mg/day  300 mg/day | | 1 (0.37%)  2 (0.73%)  28 (10.26%)  7 (2.56%)  99 (36.26%)  1 (0.37%)  1 (0.37%)  16 (5.86%)  5 (1.83%)  65 (23.81%)  1 (0.37%)  1 (0.37%)  17 (6.23%)  26 (9.52%)  1 (0.37%)  1 (0.37%)  1 (0.37%) | |
| *2021 ESC Guidelines recommendations: starting dose 12.25 mg o.d., target dose 200 mg o.d.* | | | |
| Propranolol* | | n (%) | |
| 10 mg/day  80 mg/day | | 1 (50.00%)  1 (50.00%) | |
| Betaxolol* | | n (%) | |
| 10 mg/day  20 mg/day  40 mg/day | | 2 (15.38%)  10 (76.92%)  1 (7.69%) | |
| Sotalolol* | | n (%) | |
| 40 mg/day  80 mg/day  160 mg/day  240 mg/day | | 1 (16.67%)  1 (16.67%)  3 (50.00%)  1 (16.67%) | |
| **MRA** | |  | |
| Eplerenone | | n (%) | |
| 12.5 mg/day  25 mg/day  50 mg/day  100 mg/day | | 8 (2.85%)  165 (58.72%)  106 (37.72%)  2 (0.71%) | |
| *2021 ESC Guidelines recommendations: starting dose 25 mg o.d., target dose 50 mg o.d.* | | | |
| Spironolactone | | n (%) | |
| 8.33 mg/day  12 mg/day  12.5 mg/day  25 mg/day  37.5 mg/day  50 mg/day  100 mg/day  8.33 mg/day | | 1 (0.19%)  1 (0.19%)  25 (4.68%)  330 (61.8%)  1 (0.19%)  171 (32.02%)  5 (0.94%)  1 (0.19%) | |
| *2021 ESC Guidelines recommendations: starting dose 25 mg o.d., target dose 50 mg o.d.* | | | |

- *No recommendation on the optimal dose in heart failure by the ESC 2021 Guidelines [14].
- ACE-I, angiotensin-converting enzyme inhibitors; ARB, angiotensin receptor blockers; ARNI, angiotensin receptor-neprilysin inhibitor; b.i.d., twice daily dosing; ESC, *European Society of Cardiology*; GDMT, guideline-directed medical therapy; MRA, mineralocorticoid receptor antagonists; o.d., once daily dosing.
- **Supplementary Table 3. Summary of changes performed in GDMTs excluding dapagliflozin at 6- and 12-month follow-up in FAS (class level)**

|  | Index to 6 months | 6 to 12 months |
| --- | --- | --- |
|  | n (%) | n (%) |
| **ACE-I overall changes** | **N=466** | **N=451** |
| Discontinuation | 23 (4.94%) | 17 (3.77%) |
| Dose decrease | 4 (0.86%) | 6 (1.33%) |
| Dose increase | 12 (2.58%) | 5 (1.11%) |
| Addition | 8 (1.72%) | 7 (1.55%) |
| Addition & discontinuation | - | 1 (0.22%) |
| No changes | 419 (89.91%) | 393 (87.14%) |
| **ARB overall changes** | **N=105** | **N=100** |
| Discontinuation | 5 (4.76%) | 2 (2.00%) |
| Dose decrease | 3 (2.86%) | 1 (2.00%) |
| Dose increase | 3 (2.86%) | 2 (2.00%) |
| Addition  No changes | 2 (1.9%)  91 (86.67%) | 1 (1.00%)  90 (90.00%) |
| **ARNI overall changes** | **N=460** | **N=462** |
| Discontinuation | 9 (1.96%) | 6 (1.31%) |
| Dose decrease | 9 (1.96%) | 12 (2.6%) |
| Dose increase | 21 (4.57%) | 20 (4.33%) |
| Addition | 27 (5.87%) | 14 (3.03%) |
| Addition & dose increase | 1 (0.22%) | - |
| Addition & dose decrease | 1 (0.22%) | - |
| Addition & discontinuation | - | 1 (0.22%) |
| No changes | 388 (84.35%) | 386 (83.55%) |
| **Beta-blockers overall changes** | **N=1057** | **N=1051** |
| Discontinuation | 14 (1.32%) | 14 (1.33%) |
| Dose decrease | 20 (1.89%) | 12 (1.14%) |
| Dose increase | 27 (2.55%) | 20 (1.90%) |
| Addition | 12 (16.22%) | 11 (1.05%) |
| Addition & dose decrease | 1 (1.14%) | - |
| Addition & discontinuation | - | 2 (0.19%) |
| No change | 978 (92.53%) | 936 (89.06%) |
| **MRA overall changes** | **N=826** | **N=814** |
| Discontinuation | 20 (2.42%) | 17 (2.09%) |
| Dose decrease | 12 (1.45%) | 9 (1.11%) |
| Dose increase | 10 (1.21%) | 5 (0.61%) |
| Addition | 11 (1.33%) | 12 (1.47%) |
| No changes | 769 (93.1%) | 727 (89.13%) |

- The sum of the percentages may be <100%, since treatment changes for patients lost-to-follow up between study visits were not collected.
- ACE-I, angiotensin-converting enzyme inhibitors; ARB, angiotensin receptor blockers; FAS, full analysis set; GDMT, guideline-directed medical therapy; mo, month; MRA, mineralocorticoid receptor antagonists.
- **Supplementary Table 4. Reimbursement level of dapagliflozin in HFrEF in the participating countries**

| Country | Baseline | End of study |
| --- | --- | --- |
|  | FSI: February 2022 | LPLV: December 2023 |
| Bulgaria | 75% | No change from baseline |
| Croatia | 60% | No change from baseline |
| Estonia | 75% if age <63 years  90% if age ≥63 years | No change from baseline |
| Hungary | Not reimbursed | No change from baseline |
| Latvia | Not reimbursed | 75% reimbursement from September 2023 |
| Lithuania | Not reimbursed | No change from baseline |
| Poland | 70% | No change from baseline |
| Romania | Not reimbursed, co-payment offered in a Patient Support Program | 100% reimbursement from March 2023 |
| Slovenia | 100% | No change from baseline |

- FSI, first patient in; HFrEF, heart failure with reduced ejection fraction; LPLV, last patient last visit.
