# Supplementary material for: Real-world evidence with dapagliflozin in heart failure with reduced ejection fraction in Central Eastern Europe and the Baltic region (EVOLUTION-HF CEE-BA Study)
Source: ESC Heart Fail. 2026 Mar 20;13(3):xvag085. doi: 10.1093/eschf/xvag085 (PMC13175253; doi:10.1093/eschf/xvag085)
Supplement: xvag085_Supplementary_Data [file xvag085_supplementary_data.zip › TableS1(06032026).docx]

**Supplementary Table 1. List of Ethics Committees study approval/favourable opinion numbers in participating countries**

| Country | Ethics Committee Name | Approval (number and date) |
| --- | --- | --- |
| Bulgaria | Ethics Committee for Clinical Trials  (Етична комисия по клинични  изпитвания) | EKKи 0030/19-Jan-2022 |
| Croatia | Central Ethics Commission (SEP) | 381-14-09/21-21-04 from 04-  Jan-2022 |
| Estonia | Research Ethics Committee of the  University of Tartu (UT REC) | 356/T-10 from 20-Dec-2021 |
| Hungary | Medical Reaserch Council (Egeszsegugyi  Tudomanyos Tanacs ETT TUKEB) | BMEU/187-3/2022/EKU (13-Jul-  2022) and BMEU/187-5/2022/EKU  (08-Sep-2022) |
| Latvia | Ethics Committee for Clinical Research  at Pauls Stradins Clinical University  Hospital Development Society | 240522 - 1E from 24-May-  2022 |
| Lithuania | Lietuvos bioetikos komitetas  (Lithuanian BioEthics Committee) | L-22-04/1 from 28-Apr-2022 |
| Poland | Terenowa Komisja Bioetyczna przy  Narodowym Instytucie Kardiologii | IK.NPIA.0021.29.1964/22 of  15-Mar-2022 |
| Romania | Comisia Națională de Bioetică a  Medicamentului și Dispozitivelor  Medicale (National BioEthics Committee  for Medicines and Medical Devices) | 13SNI / 16-Dec-2021 |
| Slovenia | Komisija Republike Slovenje za  medicinsko etiko | 0120-499/2021/3 from 7-Dec-  2021 and 0120-499/2021/6  from 21-Jan-2022 |
