# Supplementary material for: Real-world evidence with dapagliflozin in heart failure with reduced ejection fraction in Central Eastern Europe and the Baltic region (EVOLUTION-HF CEE-BA Study)
Source: ESC Heart Fail. 2026 Mar 20;13(3):xvag085. doi: 10.1093/eschf/xvag085 (PMC13175253; doi:10.1093/eschf/xvag085)
Supplement: xvag085_Supplementary_Data [file xvag085_supplementary_data.zip › TableS2(06032026).docx]

**Supplementary Table 2**. **Distribution of doses of GDMTs excluding dapagliflozin at index date**

| **Class or Agent** | **Dose/day** | | **Number of patients (%)** |
| --- | --- | --- | --- |
| **ACE-I** | | | |
| Enalapril | | n (%) | |
| 1.25 mg/day  5 mg/day  10 mg/day  20 mg/day | | 1 (10%)  1 (10%)  5 (50%)  3 (30%) | |
| *2021 ESC Guidelines recommendations: starting dose 2.5 mg b.i.d., target dose 10-20 mg b.i.d* | | | |
| Lisinopril | | n (%) | |
| 2.5 mg/day  5 mg/day  10 mg/day  15 mg/day  20 mg/day  40 mg/day | | 1 (5%)  2 (10%)  11 (55%)  1 (5%)  4 (20%)  1 (5%) | |
| *2021 ESC Guidelines recommendations: starting dose 2.5 mg o.d., target dose 20-35 mg o.d.* | | | |
| Ramipril | | n (%) | |
| 1.25 mg/day  2.5 mg/day  3 mg/day  3.75 mg/day  5 mg/day  7 mg/day  7.5 mg/day  10 mg/day  12.5 mg/day  20 mg/day 1 | | 13 (4.94%)  85 (32.32%)  1 (0.38%)  1 (0.38%)  105 (39.92%)  1 (0.38%)  2 (0.76%)  53 (20.15%)  1 (0.38%)  (0.38%) | |
| *2021 ESC Guidelines recommendations: starting dose 2.5 mg o.d., target dose 10 mg o.d.* | | | |
| Perindopril* | | n (%) | |
| 2 mg/day  2.5 mg/day  4 mg/day  5 mg/day  6 mg/day  7.5 mg/day  8 mg/day  9 mg/day  10 mg/day  12 mg/day | | 5 (3.6%)  24 (16.66%)  11 (7.91%)  53 (36.80%)  1 (0.72%)  2 (1.44%)  12 (8.63%)  1 (0.72%)  34 (23.61%)  1 (0.72%) | |
| Zofenopril* | | n (%) | |
| 3.75 mg/day  7.5 mg/day  15 mg/day  30 mg/day  60 mg/day | | 1 (5%)  12 (60%)  1 (5%)  1 (5%)  5 (25%) | |
| Quinapril* | | n (%) | |
| 10 mg/day | | 1 (100%) | |
| **ARB** | | | |
| Candesartan | | n (%) | |
| 4 mg/day  6 mg/day  8 mg/day  16 mg/day  24 mg/day  32 mg/day | | 4 (10%)  1 (2.5%)  16 (40%)  12 (30%)  1 (2.5%)  6 (15%) | |
| *2021 ESC Guidelines recommendations: starting dose 4 mg o.d., target dose 32 mg o.d.* | | | |
| Valsartan | | n (%) | |
| 40 mg/day  60 mg/day  80 mg/day  100 mg/day  160 mg/day  240 mg/day  320 mg/day | | 5 (17.86%)  1 (3.57%)  10 (35.71%)  1 (3.57%)  9 (32.14%)  1 (3.57%)  1 (3.57%) | |
| *2021 ESC Guidelines recommendations: starting dose 40 mg b.i.d., target dose 160 mg b.i.d.* | | | |
| Losartan | | n (%) | |
| 25 mg/day  100 mg/day | | 1 (50%)  1 (50%) | |
| *2021 ESC Guidelines recommendations: starting dose 50 mg o.d., target dose 150 mg o.d.* | | | |
| Telmisartan* | | n (%) | |
| 40 mg/day  80 mg/day  160 mg/day | | 7 (31.82%)  14 (63.64%)  1 (4.55%) | |
| Olmesartan* | | n (%) | |
| 20 mg/day  40 mg/day | | 3 (37.5%)  5 (62.5%) | |
| Irbesartan* | | n (%) | |
| 150 mg/day  300 mg/day | | 1 (33.33%)  2 (66.67%) | |
| **ARNI** | |  | |
| Sacubitril/valsartan | | n (%) | |
| 24/26 mg o.d.  24/26 mg b.i.d.  49/51 mg o.d.  49/51 mg b.i.d.  49/51 mg t.i.d.  73/77 mg o.d.  73/77 mg b.i.d.  97/103 mg o.d.  97/103 mg b.i.d. | | 19 (4.41%)  170 (39.44%)  23 (5.34%)  117 (27.15%)  1 (0.23%)  1 (0.23%)  1 (0.23%)  10 (2.32%)  89 (20.65%) | |
| *2021 ESC Guidelines recommendations: starting dose 49/51 mg b.i.d. (24/26 mg b.i.d. in selected patients), target dose 97/103 mg b.i.d* | | | |
| **Beta-blockers** | |  | |
| Bisoprolol | | n (%) | |
| 1.25 mg/day  2 mg/day  2.5 mg/day  3.75 mg/day  5 mg/day  7.5 mg/day  10 mg/day  12.5 mg/day  15 mg/day  20 mg/day | | 20 (3.95%)  1 (0.2%)  107 (21.15%)  8 (1.58%)  215 (42.49%)  31 (6.13%)  108 (21.34%)  1 (0.2%)  9 (1.78%)  6 (1.19%) | |
| *2021 ESC Guidelines recommendations: starting dose 1.25 mg o.d., target dose 10 mg o.d.* | | | |
| Carvedilol | | n (%) | |
| 3.125 mg/day  6.25 mg/day  12.5 mg/day  13 mg/day  18.625 mg/day  18.75 mg/day  25 mg/day  37.5 mg/day  50 mg/day  62.5 mg/day  100 mg/day | | 1 (0.65%)  17 (11.11%)  42 (27.45%)  1 (0.65%)  1 (0.65%)  5 (3.27%)  41 (26.8%)  11 (7.19%)  32 (20.92%)  1 (0.65%)  1 (0.65%) | |
| *2021 ESC Guidelines recommendations: starting dose 3.125 mg b.i.d., target dose 25 mg b.i.d. (target dose 50 mg b.i.d. if >85 kg).* | | | |
| Nebivolol | | n (%) | |
| 1.25 mg/day  2.5 mg/day  5 mg/day  7.5 mg/day  10 mg/day | | 2 (2.2%)  20 (21.98%)  62 (68.13%)  1 (1.1%)  6 (6.59%) | |
| *2021 ESC Guidelines recommendations: starting dose 1.25 mg o.d., target dose 10 mg o.d.* | | | |
| Metoprolol succinate (CR/XL) | | n (%) | |
| 12.5 mg/day  23.75 mg/day  25 mg/day  47.5 mg/day  50 mg/day  62.5 mg/day  71.25 mg/day  75 mg/day  95 mg/day  100 mg/day  125 mg/day  142.5 mg/day  150 mg/day  200 mg/day  225 mg/day  250 mg/day  300 mg/day | | 1 (0.37%)  2 (0.73%)  28 (10.26%)  7 (2.56%)  99 (36.26%)  1 (0.37%)  1 (0.37%)  16 (5.86%)  5 (1.83%)  65 (23.81%)  1 (0.37%)  1 (0.37%)  17 (6.23%)  26 (9.52%)  1 (0.37%)  1 (0.37%)  1 (0.37%) | |
| *2021 ESC Guidelines recommendations: starting dose 12.25 mg o.d., target dose 200 mg o.d.* | | | |
| Propranolol* | | n (%) | |
| 10 mg/day  80 mg/day | | 1 (50%)  1 (50%) | |
| Betaxolol* | | n (%) | |
| 10 mg/day  20 mg/day  40 mg/day | | 2 (15.38%)  10 (76.92%)  1 (7.69%) | |
| Sotalolol* | | n (%) | |
| 40 mg/day  80 mg/day  160 mg/day  240 mg/day | | 1 (16.67%)  1 (16.67%)  3 (50%)  1 (16.67%) | |
| **MRA** | |  | |
| Eplerenone | | n (%) | |
| 12.5 mg/day  25 mg/day  50 mg/day  100 mg/day | | 8 (2.85%)  165 (58.72%)  106 (37.72%)  2 (0.71%) | |
| *2021 ESC Guidelines recommendations: starting dose 25 mg o.d., target dose 50 mg o.d.* | | | |
| Spironolactone | | n (%) | |
| 8.33 mg/day  12 mg/day  12.5 mg/day  25 mg/day  37.5 mg/day  50 mg/day  100 mg/day  8.33 mg/day | | 1 (0.19%)  1 (0.19%)  25 (4.68%)  330 (61.8%)  1 (0.19%)  171 (32.02%)  5 (0.94%)  1 (0.19%) | |
| *2021 ESC Guidelines recommendations: starting dose 25 mg o.d., target dose 50 mg o.d.* | | | |

*No recommendation on the optimal dose in heart failure by the ESC 2021 Guidelines [14].

ACE-I, angiotensin-converting enzyme inhibitors; ARB, angiotensin receptor blockers; ARNI, angiotensin receptor-neprilysin inhibitor; b.i.d., twice daily dosing; ESC, *European Society of Cardiology*; GDMT, guideline-directed medical therapy; MRA, mineralocorticoid receptor antagonists; o.d., once daily dosing.
