# Supplementary material for: Real-world evidence with dapagliflozin in heart failure with reduced ejection fraction in Central Eastern Europe and the Baltic region (EVOLUTION-HF CEE-BA Study)
Source: ESC Heart Fail. 2026 Mar 20;13(3):xvag085. doi: 10.1093/eschf/xvag085 (PMC13175253; doi:10.1093/eschf/xvag085)
Supplement: xvag085_Supplementary_Data [file xvag085_supplementary_data.zip › TableS3(06032026).docx]

**Supplementary Table 3. Summary of changes performed in GDMTs excluding dapagliflozin at 6- and 12-month follow-up in FAS (class level)**

|  | Index to 6 months | 6 to 12 months |
| --- | --- | --- |
|  | n (%) | n (%) |
| **ACE-I overall changes** | **N=466** | **N=451** |
| Discontinuation | 23 (4.94%) | 17 (3.77%) |
| Dose decrease | 4 (0.86%) | 6 (1.33%) |
| Dose increase | 12 (2.58%) | 5 (1.11%) |
| Addition | 8 (1.72%) | 7 (1.55%) |
| Addition & discontinuation | - | 1 (0.22%) |
| No changes | 419 (89.91%) | 393 (87.14%) |
| **ARB overall changes** | **N=105** | **N=100** |
| Discontinuation | 5 (4.76%) | 2 (2%) |
| Dose decrease | 3 (2.86%) | 1 (2%) |
| Dose increase | 3 (2.86%) | 2 (2%) |
| Addition  No changes | 2 (1.9%)  91 (86.67%) | 1 (1%)  90 (90%) |
| **ARNI overall changes** | **N=460** | **N=462** |
| Discontinuation | 9 (1.96%) | 6 (1.31%) |
| Dose decrease | 9 (1.96%) | 12 (2.6%) |
| Dose increase | 21 (4.57%) | 20 (4.33%) |
| Addition | 27 (5.87%) | 14 (3.03%) |
| Addition & dose increase | 1 (0.22%) | - |
| Addition & dose decrease | 1 (0.22%) | - |
| Addition & discontinuation | - | 1 (0.22%) |
| No changes | 388 (84.35%) | 386 (83.55%) |
| **Beta-blockers overall changes** | **N=1057** | **N=1051** |
| Discontinuation | 14 (1.32%) | 14 (1.33%) |
| Dose decrease | 20 (1.89%) | 12 (1.14%) |
| Dose increase | 27 (2.55%) | 20 (1.9%) |
| Addition | 12 (16.22%) | 11 (1.05%) |
| Addition & dose decrease | 1 (1.14%) | - |
| Addition & discontinuation | - | 2 (0.19%) |
| No change | 978 (92.53%) | 936 (89.06%) |
| **MRA overall changes** | **N=826** | **N=814** |
| Discontinuation | 20 (2.42%) | 17 (2.09%) |
| Dose decrease | 12 (1.45%) | 9 (1.11%) |
| Dose increase | 10 (1.21%) | 5 (0.61%) |
| Addition | 11 (1.33%) | 12 (1.47%) |
| No changes | 769 (93.1%) | 727 (89.13%) |

The sum of the percentages may be <100%, since treatment changes for patients lost-to-follow up between study visits were not collected.

ACE-I, angiotensin-converting enzyme inhibitors; ARB, angiotensin receptor blockers; FAS, full analysis set; GDMT, guideline-directed medical therapy; mo, month; MRA, mineralocorticoid receptor antagonists.
