# Supplementary material for: Real-world evidence with dapagliflozin in heart failure with reduced ejection fraction in Central Eastern Europe and the Baltic region (EVOLUTION-HF CEE-BA Study)
Source: ESC Heart Fail. 2026 Mar 20;13(3):xvag085. doi: 10.1093/eschf/xvag085 (PMC13175253; doi:10.1093/eschf/xvag085)
Supplement: xvag085_Supplementary_Data [file xvag085_supplementary_data.zip › TableS4(06032026).docx]

**Supplementary Table 4. Reimbursement level of dapagliflozin in HFrEF in the participating countries**

| Country | Baseline | End of study |
| --- | --- | --- |
|  | FSI: February 2022 | LPLV: December 2023 |
| Bulgaria | 75% | No change from baseline |
| Croatia | 60% | No change from baseline |
| Estonia | 75% if age <63 years  90% if age ≥63 years | No change from baseline |
| Hungary | Not reimbursed | No change from baseline |
| Latvia | Not reimbursed | 75% reimbursement from September 2023 |
| Lithuania | Not reimbursed | No change from baseline |
| Poland | 70% | No change from baseline |
| Romania | Not reimbursed, co-payment offered in a Patient Support Program | 100% reimbursement from March 2023 |
| Slovenia | 100% | No change from baseline |

FSI, first patient in; HFrEF, heart failure with reduced ejection fraction; LPLV, last patient last visit.
