# Supplementary figures and images for: Real-world evidence with dapagliflozin in heart failure with reduced ejection fraction in Central Eastern Europe and the Baltic region (EVOLUTION-HF CEE-BA Study)
Source: ESC Heart Fail. 2026 Mar 20;13(3):xvag085. doi: 10.1093/eschf/xvag085 (PMC13175253; doi:10.1093/eschf/xvag085)

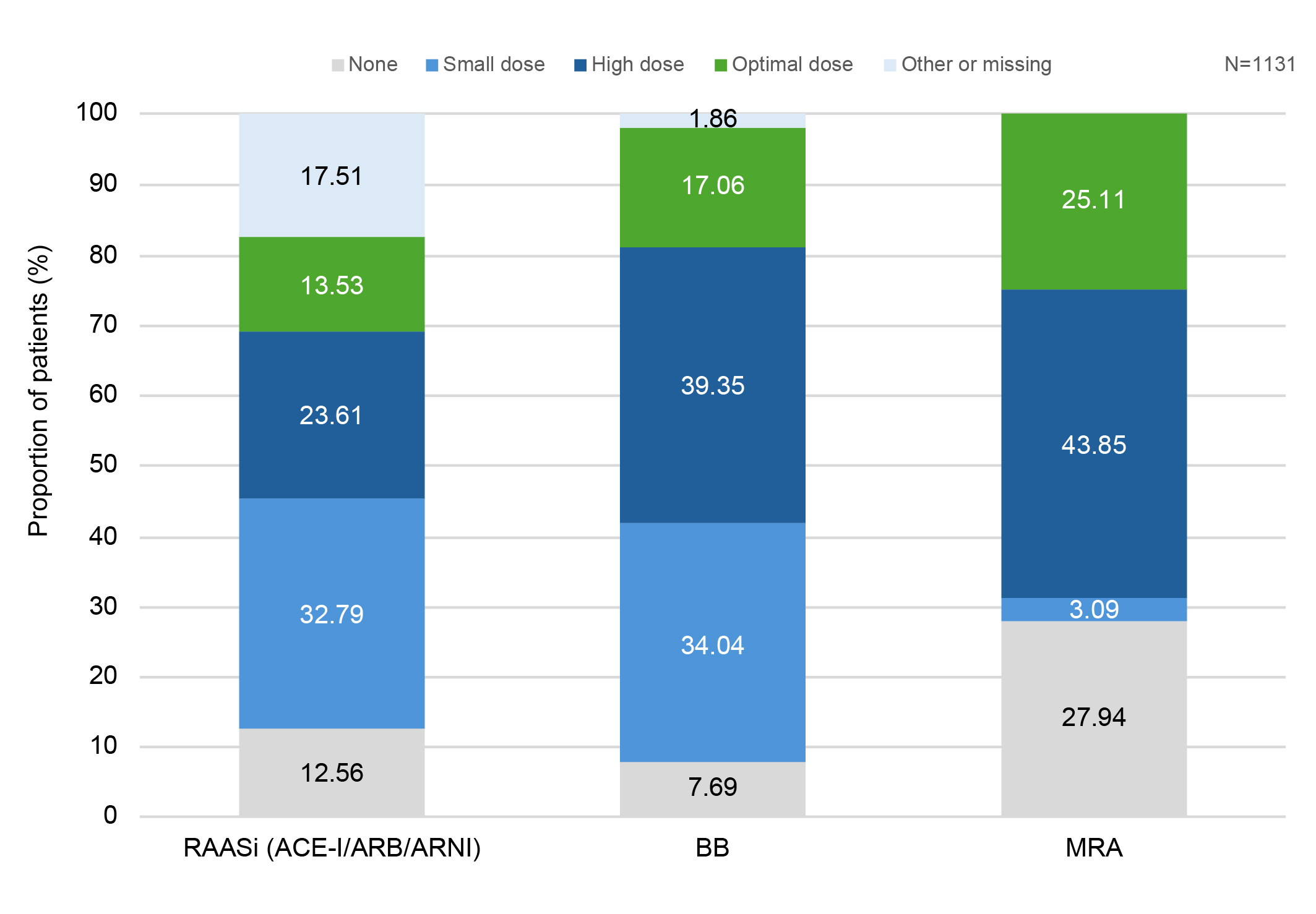

Supplement: xvag085_Supplementary_Data [file xvag085_supplementary_data.zip › Suppl Figure 1_06032026.jpg]
